# Supplementary material for: Microwave Irradiation as a Powerful Tool for the Preparation of n-Type Benzotriazole Semiconductors with Applications in Organic Field-Effect Transistors
Source: Molecules. 2022 Jul 6;27(14):4340. doi: 10.3390/molecules27144340 (PMC9323175; doi:10.3390/molecules27144340)
Supplement: Supplementary file 1 [file molecules-27-04340-s001.zip › molecules-1767744-supplementary.pdf]

Supplementary Materials

Microwave Irradiation as a Powerful Tool for the Preparation of n-type Benzotriazole Semiconductors with Applications in Organic Field-Effect Transistors

Iván Torres-Moya [1,*], Alexandra Harbuzaru [2], Beatriz Donoso [1], Pilar Prieto [1], Rocío Ponce Ortiz [2,*] and Ángel Díaz-Ortiz [1,*]

[1] Department of Inorganic, Organic Chemistry and Biochemistry, Faculty of Science and Chemical Technologies, University of Castilla-La Mancha-IRICA, 13071 Ciudad Real, Spain; beatriz.donoso@uclm.es (B.D.); mariapilar.prieto@uclm.es (P.P.)

[2] Department of Physical Chemistry, Faculty of Sciences, University of Málaga, Campus of Teatinos s/n., 29071 Málaga, Spain; harbuzaru@uma.es (A.H.)

* Correspondence: ivan.torresmoya@uclm.es (I.T.-M.); rocioponce@uma.es (R.P.O.); angel.diaz@uclm.es (Á.D.-O.)

INDEX

1. **Experimental section -- 2**
2. **Previous D-A-D reported benzotriazoles by our research group--------- 4**
3. **NMR spectra -- 5**
4. **Cartesian coordinates of benzotriazole 1 --------------------------------------- 6**

1. Experimental section

Synthesis of (*E*)-1-(3,5-bis(trifluoromethyl)phenyl)-2-(2-nitrophenyl)diazene (**4**)

A mixture of 2-nitrosonitrobenzene **2** (0.100 g, 0.66 mmol), the corresponding aniline **3** (0.151 g, 0.66 mmol) and 1 mL of acetic acid was added to a dried microwave vessel. The vessel was then closed and irradiated with microwave irradiation at 60 °C for 20 min. After this time, the reaction mixture was purified by chromatography, employing hexane/ethyl acetate (9:1) as eluent, to give a red solid (0.225 g, 94%). The NMR spectra and MS data for which agreed with those reported previously by our research group.

2-(3,5-bis(trifluoromethyl)phenyl)-2*H*-benzo[*d*][1,2,3]triazole (**6**) A mixture of azo derivative **4** (0.100 g, 0.28 mmol), formamidinesulfinic acid (0.156 g, 1.45 mmol), 0.5 mL of NaOH, and 0.75 mL of *tert*-butanol was added to a dried microwave vessel. The vessel was then closed and irradiated with microwave irradiation at 80 °C for 30 min. After this time the reaction mixture was cooled, put into a mixture of ice/H_2O (20 mL) and the solid obtained was filtered and washed to give a pale yellow solid (0.072 g, 78 %) without any need for further purification. The NMR and MS data for this compound agreed with those reported previously by our research group.

2-(3,5-bis(trifluoromethyl)phenyl)-4,7-dibromo-2*H*-benzo[*d*][1,2,3]triazole (**7**): In an open microwave vessel, benzotriazole **6** (0.100 g, 0.30 mmol) was carefully mixed and stirred with 1 mL of acetic acid and 1 mL of bromine, irradiating at 100 °C for 30 min with microwave irradiation. The mixture was then cooled and poured into a mixture of ice/H_2O (50 mL) to precipitate a brown solid, which was purified by column chromatography using hexane/ethyl acetate (9:1) as eluent. The resulting pale brown solid (0.118 g, 80%) gave satisfactory NMR and MS data in agreement with those reported previously by our research group.

Synthesis of 2-(3,5-bis(trifluoromethyl)phenyl)-4,7-dibromo-5,6-dinitro-2*H*-benzo[*d*][1,2,3]triazole (**8**).

Concentrated nitric acid (1 mL) and concentrated sulfuric acid (1 mL) were introduced into a closed microwave vessel at 0 °C. Benzotriazole **7** (0.250 g, 0.51 mmol) was then added, irradiating for 10 min at 60 °C with microwave irradiation. The mixture was then cooled, poured into a mixture of ice/H_2O (50 mL) and extracted with diethyl ether (2 x 30 mL). The resulting organic layer

was washed twice with H_2O (30 mL) and dried over $MgSO_4$. The solvent was removed under vacuum and the crude mixture purified by column chromatography on silica gel, using hexane/ethyl acetate (9:1) as eluent. The resulting pale brown solid (0.272 g, 92%) gave satisfactory NMR and MS data in agreement with those reported previously by our research group.

Synthesis of 2-(3,5-bis(trifluoromethyl)phenyl)-4,7-dibromo-2*H*-benzo[*d*][1,2,3]triazole-5,6-diamine (**9**)

First of all, CuNPs (15 mg) and 1 ml of glycerol were added to a closed microwave vessel and were sonicated until form a dark dispersion. Dinitrobenzotriazole **8** (0.100 g, 0.172 mmol) and KOH (0.019 g, 0.344 mmol) were then added and the reaction performed at 130 °C for 15 min under microwave irradiation. The crude reaction mixture was then cooled to room temperature and filtered to remove the CuNPs. At that point, 20 mL of water was added to the mixture, which was then extracted with ethyl acetate (2 x 20 mL). HCl 20% (20 mL) was added to the organic phase and, after extraction, the aqueous phase was basified with NaOH (0.01 M), extracted with ethyl acetate (3 x 50 mL), dried over magnesium sulfate and filtered under vacuum to give the pure product as a brown solid (0.089 g, 100%) that did not require any further purification. The NMR and MS data for this compound were in agreement with those reported previously by our research group.

Synthesis of 12-(3,5-bis(trifluoromethyl)phenyl)-10,14-dibromo-12*H*-dibenzo[*a,c*][1,2,3]triazolo[4,5-*i*]phenazine (**11**)

Diketone **10** (0.044 g, 0.211 mmol) and diamino derivative **9** (0.100 g, 0.192 mmol) were added to a microwave vessel, followed by 1 mL of a mixture of ethanol/glacial acetic acid (8:2). The vessel was closed and the mixture stirred well while being heated to 100 °C for 30 minutes under microwave irradiation. The crude reaction mixture was then cooled, the solvents removed and the dark red solid obtained was washed with hot glacial acetic acid (3 x 10 mL) and ice water (2 x 10 mL). The resulting dark red solid (0.118 g, 90%) gave satisfactory NMR and MS data in agreement with those reported previously by our research group.

2. *Previous D-A-D reported benzotriazoles by our research group*

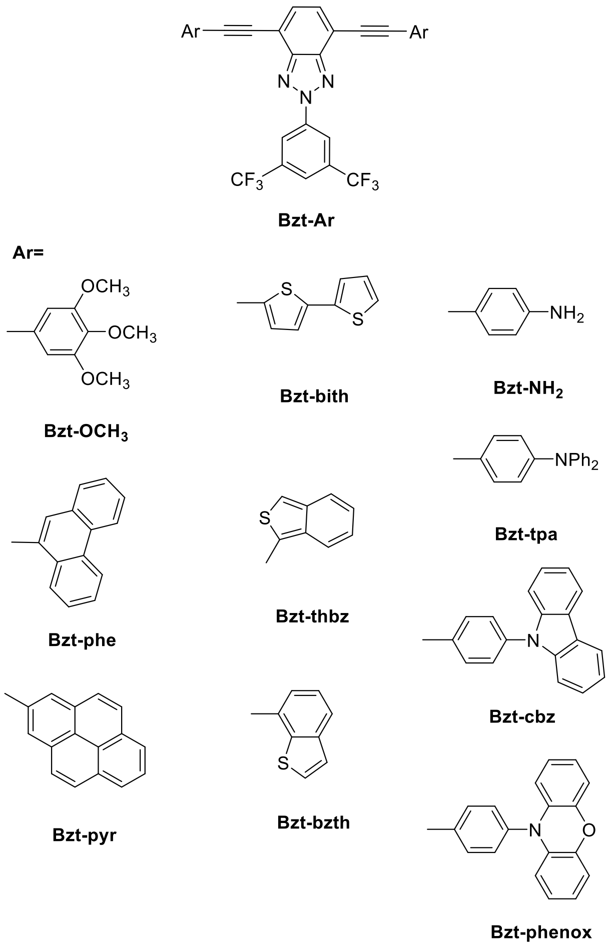

Table S1. OFET electrical data for devices fabricated with organic semiconductors **Bzt-Ar** measured in vacuum.

**Semiconductor (HMDS 90 °C)**	**μ_h ($cm^2V^{-1}s^{-1}$)**	**V_T (V)**	**I_{ON}/I_{OFF}**
**Bzt-phe**	2.69×10^{-4}	–57	2×10^3
**Bzt-pyr**	3.31×10^{-5}	–40	1×10^2
**Bzt-bith**	2.89×10^{-5}	-47	3×10^2
**Bzt-thbz**	1.80×10^{-4}	–69	8×10^3
**Bzt-bzth**	3.76×10^{-5}	–13	4×10^2
**Bzt-tpa**	1.21×10^{-4}	–38	2×10^2
**Bzt-cbz**	2.02×10^{-5}	–58	3×10^2
**Bzt-phenox**	2.04×10^{-5}	–40	5×10^2

3. NMR spectra

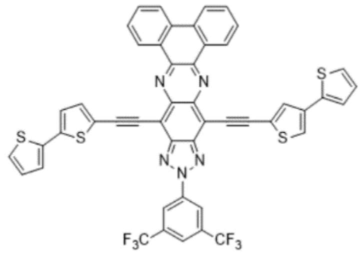

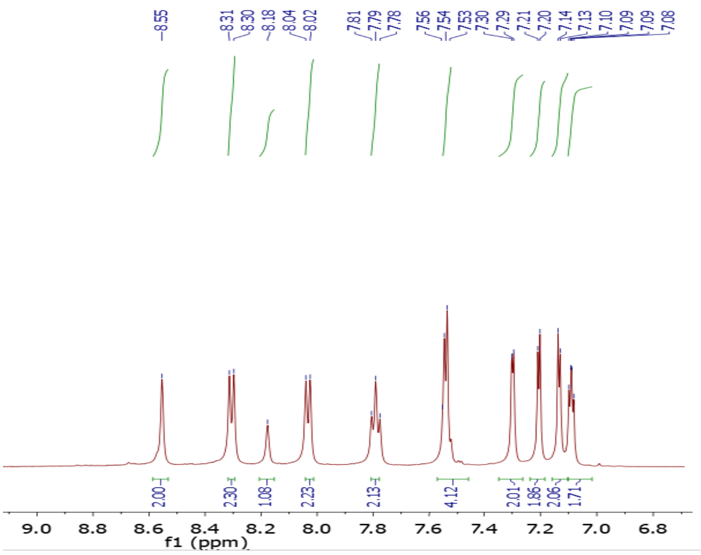

Figure S1. ^{1}H-NMR spectrum of compound **1** in DMSO.

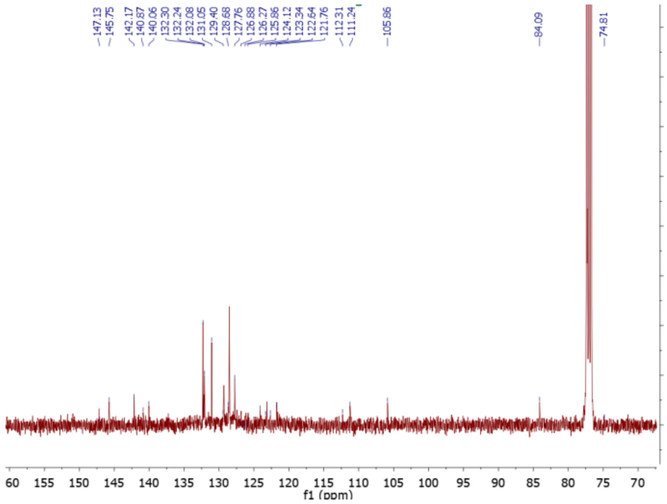

Figure S2. ^{13}C-NMR spectrum of compound **1** in DMSO.

4. *Cartesian coordinates of benzotriazole 1*

Table S2. Cartesian coordinates for optimized geometry of **1.**

Center Number	Atomic Number	Atomic Type	Coordinates (Angstroms) X	Y	Z
1	6	0	-0.716046	-0.159935	0.002931
2	6	0	0.716052	-0.159922	0.002943
3	6	0	1.480682	1.032556	0.007481
4	6	0	0.725766	2.243622	0.012361
5	6	0	-0.725807	2.243608	0.012345
6	6	0	-1.480699	1.032527	0.007453
7	6	0	0.000038	-3.551474	-0.006525
8	6	0	1.216945	-4.235365	-0.012730
9	6	0	-1.216856	-4.235392	-0.012612
10	6	0	1.203983	-5.628484	-0.024616
11	1	0	2.145473	-3.679958	-0.018648
12	6	0	-1.203865	-5.628510	-0.024501
13	1	0	-2.145397	-3.680004	-0.018444
14	6	0	0.000066	-6.333740	-0.031886
15	1	0	0.000075	-7.416387	-0.059066
16	6	0	-2.509496	-6.382723	0.023886
17	6	0	2.509634	-6.382671	0.023630
18	9	0	-2.885618	-6.630751	1.298113
19	9	0	-3.509224	-5.689446	-0.558527
20	9	0	-2.416178	-7.575586	-0.601456
21	9	0	2.416289	-7.575507	-0.601759
22	9	0	2.885864	-6.630750	1.297815
23	9	0	3.509300	-5.689346	-0.558833
24	7	0	1.140059	-1.440775	-0.001554
25	7	0	-1.140026	-1.440796	-0.001557
26	7	0	1.401725	3.417813	0.017009
27	7	0	-1.401790	3.417785	0.016968
28	6	0	0.722043	4.549985	0.021417
29	6	0	-0.722130	4.549971	0.021391
30	6	0	1.449700	5.819610	0.026432
31	6	0	-1.449811	5.819581	0.026367
32	6	0	0.737386	7.045877	0.031478
33	6	0	-0.737522	7.045862	0.031443
34	6	0	2.856625	5.816244	0.026184
35	6	0	1.487711	8.238670	0.036299
36	6	0	3.567735	7.004606	0.030938
37	6	0	2.875168	8.222350	0.036065
38	6	0	-2.856737	5.816187	0.026051
39	6	0	-1.487871	8.238640	0.036222
40	6	0	-2.875327	8.222293	0.035920
41	6	0	-3.567870	7.004535	0.030765
42	1	0	-0.983970	9.197674	0.040204
43	1	0	-3.422027	9.160664	0.039659
44	1	0	-4.653522	6.992245	0.030419
45	1	0	-3.360076	4.856200	0.021937
46	1	0	3.359984	4.856267	0.022090

47	1	0	4.653387	6.992337	0.030646
48	1	0	3.421848	9.160732	0.039834
49	1	0	0.983791	9.197693	0.040262
50	6	0	-2.882487	1.009836	0.007005
51	6	0	2.882472	1.009890	0.007049
52	6	0	-4.102764	0.945828	0.006755
53	6	0	4.102750	0.945904	0.006809
54	7	0	0.000022	-2.130713	-0.004276
55	6	0	5.494937	0.849999	0.004954
56	6	0	6.437360	1.867335	0.021774
57	16	0	6.295197	-0.720851	-0.023619
58	6	0	7.768245	1.402752	0.021766
59	1	0	6.155698	2.912857	0.040791
60	6	0	7.881389	0.022557	0.003335
61	1	0	8.629950	2.060054	0.048586
62	6	0	9.080112	-0.785611	0.001275
63	6	0	9.209237	-2.146497	0.191891
64	16	0	10.658497	-0.062475	-0.273091
65	6	0	10.553354	-2.605176	0.125257
66	1	0	8.363116	-2.795365	0.388185
67	6	0	11.447952	-1.598095	-0.114562
68	1	0	10.842812	-3.641286	0.255808
69	1	0	12.522796	-1.661333	-0.209725
70	6	0	-5.494948	0.849912	0.004906
71	6	0	-6.437375	1.867244	0.021733
72	16	0	-6.295197	-0.720940	-0.023676
73	6	0	-7.768259	1.402652	0.021724
74	1	0	-6.155717	2.912767	0.040748
75	6	0	-7.881391	0.022457	0.003281
76	1	0	-8.629972	2.059942	0.048533
77	6	0	-9.080109	-0.785722	0.001202
78	6	0	-9.209192	-2.146653	0.191524
79	16	0	-10.658532	-0.062541	-0.272803
80	6	0	-10.553315	-2.605330	0.124964
81	1	0	-8.363033	-2.795553	0.387546
82	6	0	-11.447953	-1.598202	-0.114512
83	1	0	-10.842747	-3.641473	0.255312
84	1	0	-12.522808	-1.661431	-0.209553
